# Large‐Area Perovskite Film Prepared by New FFASE Method for Stable Solar Modules Having High Efficiency under Both Outdoor and Indoor Light Harvesting

**DOI:** 10.1002/advs.202205967

**Published:** 2023-01-16

**Authors:** Chien‐Hung Chiang, Chun‐Guey Wu

**Affiliations:** ^1^ Research Center for New Generation Photovoltaics National Central University ROC Jhong‐Li 32001 Taiwan; ^2^ Department of Chemistry, National Central University ROC Jhong‐Li 32001 Taiwan

**Keywords:** freely falling anti‐solvent extraction, perovskite solar module, post treatment

## Abstract

High‐quality perovskite film is deposited on a 30 cm × 40 cm LiCoO_2_‐coated ITO/glass via newly developed freely falling anti‐solvent extraction (FFASE) method followed by post watervapor annealing in an ambient atmosphere. Perovskite solar modules (PSMs, active area of 25.2 cm^2^ with mask) based on this high‐quality film achieve the highest efficiency of 16.04 and 30.76% under 1 sun (100 mW cm^−2^) and 945 lux fluorescent light illumination, respectively. The encapsulated PSMs are stable at −20 to 80 °C thermal cycling and keep high efficiency at temperature as low as −20 °C and as high as 80 °C. When the encapsulated PSM is heated at 85 °C and 85% relative humidity under room lighting or heated at 60 °C under AM1.5 (100 mW cm^−2^) illumination for 1000 h, loses only ≈8% of its original efficiency. The high stability of PSMs is due to very high quality perovskite absorber being used. The underlying concept of the FFASE method for extracting the solvent from the large‐area perovskite precursor film is that the whole precursor film contacts with the fresh anti‐solvent during the crystallization stage.

## Graphical abstract

High‐quality perovskite film is deposited on a 30 cm × 40 cm LiCoO_2_‐coated ITO/glass via newly developed freely falling anti‐solvent extraction followed by post water vapor annealing in an ambient atmosphere. Perovskite solar modules based on this high‐quality film achieve the power conversion efficiency of 16.04% under 1 sun and very stable at temperature between −20 and 80 °C.

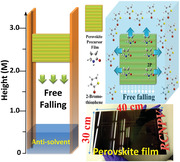

## 1 Introduction

Perovskite solar cell (PSC) is a photovoltaic device using perovskite material as a photoabsorber. PSC is regarded as one of the most promising new generation photovoltaic techniques due to several reasons, such as low‐cost solution process, various cell architectures, using small amount and diversity materials as well as high power conversion efficiency (PCE).^[^ ^1^, ^2^, ^3^ ^]^ The highest PCE of the regular type (n‐i‐p) PSC is closed to 26%^[^ ^4^ ^]^ and the low‐temperature processed inverted type (p‐i‐n) PSC has also achieved the highest PCE of over 25%,^[^ ^5^ ^]^ which is comparable with the commercialized thin‐film compound semiconductor solar cells.^[^ ^6^ ^]^ Nevertheless, those PCE data are all based on the cell with the active area of typical 0.02–1 cm^2^ and the power output is only 0.4–25 mW even under 1 sun (AM 1.5G) illumination. Therefore, to successfully commercialize PSCs, methods for fabricating high‐efficiency, stable large‐scale solar modules should be developed. High‐efficiency perovskite solar module (PSM) critically depends on the ability to fabricate large‐sized, high‐quality perovskite films, most presumably through cost‐effective solution processes. Nevertheless, owing to the low entropy and low activation energy for the formation of the ionic perovskite crystal, controlling over the film morphology of scalable‐coated perovskite films is very evasive.

Carlo and co‐workers^[^ ^7^ ^]^ are the first group to assemble PSM in 2014. They used a spin‐coating method to prepare CH_3_NH_3_PbI_3−_ *_x_* Cl*_x_* film and the corresponding regular SPM with active area of 16.8 cm^2^ achieved the highest PCE of 5.1% with a low fill factor (FF) of 57%, due to the poor morphology of the perovskite absorber. To improve the photovoltaic performance of PSMs, the first and foremost requirement is to prepare large‐area, high‐quality perovskite film with well‐control in morphology, uniformity, coverage, grain size, crystallinity, crystal orientation, etc. Several processing strategies have successfully been used to prepare high‐quality perovskite film, such as anti‐solvent bathing,^[^ ^8^, ^9^ ^]^ anti‐solvent extraction,^[^ ^10^, ^11^ ^]^ hot casting,^[^ ^12^, ^13^, ^14^, ^15^ ^]^ forced gas flow,^[^ ^16^, ^17^, ^18^, ^19^ ^]^ vacuum‐assisted drying,^[^ ^20^, ^21^, ^22^, ^23^ ^]^ etc. The underlying principle of these methods is to form a homogeneous precursor film then rapidly and uniformly remove the solvent from the solid‐state precursor films to control the even crystal growth. However, during the formation of crystalline perovskite film, the steps involving solvent removal, nucleation, and grain growth often occur with a significant overlap. It is very challenging to control these complex processes exactly in a scalable deposition. Therefore, scaling up from the small‐ to large‐area perovskite film deposition (even based on the same strategy) requires reevaluation/redesign of many parameters associated with the film preparation processes. For example, casting uniform, large‐area perovskites thin films with the common used spin‐coating method (for high‐quality small‐area film) is not trivial. Perovskite is an ionic compound therefore its solution exhibits low viscosity, leads to rapid formation of perovskite crystals. As the crystals grow the material is pulled away from the regions surrounding the crystals and then the openings in the film are formed. These openings have a serious deleteriously impact on the photovoltaic properties of the corresponding devices. To prepare large‐area perovskite film without those openings certainly is not as easy as for fabricating small‐area film. As a result, at present, the certified PSM efficiency (<18% for 55 cells with the aperture area of 804 cm^2^)^[^ ^24^ ^]^ is still far away from that (25.7%) of the state‐of‐the‐art laboratory‐scale PSCs.^[^ ^6^, ^24^, ^25^ ^]^ There are even more challenges/limitations for scalable coating of high‐quality perovskite film at the product‐relevant scale (e.g., 1 m^2^ area). Therefore carefully designing the specific perovskite inks (materials/compositions), coating methods, film crystallization techniques, even using “greener” solution chemistry and processing, etc. are necessary. Moreover, scalable coating of carrier transporting layers should also be concerned to ensure the feasibility for perovskite film deposition (or depositing on perovskite) and effective carrier extraction in PSM. Furthermore, to be commercialized, the stability of PSM should consider to pass the IEC61215 standard (the most used international standard for mature photovoltaic technologies), which was not reported for most of PSM studies. Here, we present the strategy for fabricating high‐quality, large‐area (30 cm × 40 cm) perovskite film for inverted PSMs (light first passes through the hole‐selective contact) based on spin‐coating, anti‐solvent extraction and post film treatment processes. The 10 cm × 10 cm inverted PSMs (diced from the 30 cm × 40 cm substrate) exhibit the highest efficiency of 16% and 30% at 1 sun (100 mW cm^−2^) and 1000 lux T5 light illumination, respectively. The PSMs are also stable at 85 °C, 85% relative humidity under room lighting, 60 °C under 1 sun illumination, and thermal cycling between −20 and 80 °C under vacuum. The results demonstrate the potential for scaling up the fabrication of perovskite films for PSMs using the simple and reproducible processes.

## 2 Results and Discussion

**Figure 1:**
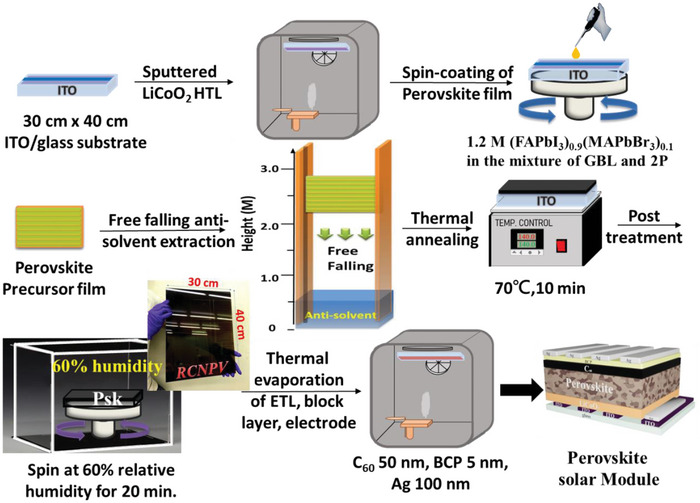
Schematic representation of the processes for fabricating perovskite solar module.

The fabrication processes for the inverted perovskite solar module are illustrated in **Figure** **1**. According to the solar efficiency tables 58 published in 2021,^[^ ^24^ ^]^ perovskite solar submodule with the highest verified efficiency has a designated illumination area (da) of 804 cm^2^. Therefore, ITO/glass substrate with an area (30 cm × 40 cm) higher than 804 cm^2^ was chosen as an electrode in this study. To be able to fabricate high‐efficiency perovskite solar modules, every step, which may different from those for assembling small cells, should be concerned carefully. For PSM with an inverted architecture, the first step is to deposit large‐area hole transporting layer (HTL) on ITO substrate. However, when PEDOT:PSS (the material generally used to make hole transporting layer in an inverted PSCs) aqueous solution was spin‐coated on a 30 cm × 40 cm ITO/glass substrate, a noncontinuous film was formed, which can be seen by naked eye. This probably is due to the lower viscosity of PEODT:PSS aqueous solution and the solvent (H_2_O) vaporized quickly during spinning. Furthermore, it is also known that it is difficult to deposit high‐quality, large‐area perovskite thin film on polymeric (such as PEDOT:PSS) surface which is an event intimately associated with the crystallization kinetics of ionic perovskites.^[^ ^25^, ^26^, ^27^, ^28^, ^29^ ^]^ As a result, PSM (10 cm × 10 cm, diced from 30 cm × 40 cm substrate) based on PEDOT:PSS HTL, (FAI)_0.9_(PbI_2_)_0.9_(MABr)_0.1_(PbBr_2_)_0.1_ absorber, and C_60_ electron transporting layer (ETL) has very low PCE of 1.90% (the *I–V* curve and photovoltaic parameters were displayed in Figure S1, Supporting Information). To solve this problem, sputtered continuous LiCoO_4_ film^[^ ^30^ ^]^ with super hydrophilicity was deposited on a 30 cm × 40 cm ITO/glass substrate to be a hole transporting layer.

**Figure 2:**
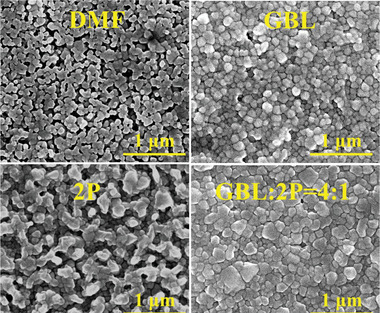
SEM images of the large area perovskite films prepared with four different solvents for precursor solutions.

For considering the compositions of perovskite ink, it was shown that mixed cations and mixed halide perovskites have better resistance to moisture and heat than MAPbI_3_.^[^ ^31^ ^]^ Therefore FAI, PbI_2_, MABr, and PbBr_2_ (the mixtures of FA, MA, Br, and I) were used as the reagents for perovskite absorbers. Various molar ratios of these four components were test using the small cells to find out the best molar ratio of these four perovskite sources is 0.9:0.9:0.1:0.1 (with the stoichiometry of FAI)_0.9_(PbI_2_)_0.9_(MABr)_0.1_(PbBr_2_)_0.1_) when one‐step spin‐coating combined with an anti‐solvent engineering method^[^ ^8^ ^]^ were used to prepare perovskite films. To deposit large‐area, high‐quality perovskite film on LiCoO_4_ HTL using one‐step spin‐coating method, the solvent for the perovskite precursor solution also need to be fine‐tuned. Guo et al.^[^ ^32^ ^]^ presented recently a review article to discuss how to control the crystallization dynamics of the large‐area perovskite film. They highlight that independent of the coating method, creating intermediate phases to decouple the overlap of the solution coating, nucleation, and crystal growth is essential to realize homogeneous deposition of perovskite films, especially large‐area perovskite thin film. It was also known that the formation of Lewis acid‐base adduct (such as PbI_2_:DMSO (dimethyl sulfoxide) adduct^[^ ^33^ ^]^) can control the crystallization kinetics of perovskite. Therefore, four solvents for the perovskite precursor, such as the common used dimethylformamide (DMF), gamma‐butyrolactone (GBL), 2‐pyrrolidinone (2P) (very low vapor pressure nontoxic liquid discovered by our group to replace DMSO),^[^ ^34^ ^]^ and their mixture, were investigated. The underlying concept of the solvent chosen concerned also the boiling point of the solvents (besides forming acid‐base adduct) to keep some solvent molecules in perovskite precursor film before applying an anti‐solvent since a wide processing window of the perovskite precursor film is needed to ensure reproducible and scalable fabrication of large‐area homogeneous perovskite film.^[^ ^14^ ^]^ Low‐toxic 2P has very high boiling point and can form PbI_2_:2P adduct with PbI_2_ to slow down the perovskite crystallization kinetics.^[^ ^34^ ^]^ Using a mixture of GBL and 2P with a volume ratio of 4:1, a process window of more than 10 min was achieved. Anti‐solvent extraction (extracting solvent of the precursor (wet) film with a liquid cannot dissolve perovskite) was well known to be an effective method to prepare high‐quality, small‐area perovskite films.^[^ ^8^, ^35^ ^]^ Applying anti‐solvent to the perovskite precursor film involves the solvent extraction and nucleation two steps (may also involve the further crystal growth). It is well accepted that the morphology of the perovskite film depends strongly on the rates of these steps, which are closely related to the constantly changing concentrations of the precursor wet film by the evaporation of the solvent. The crystallization protocol based on anti‐solvent dripping of the spin‐coated film is to add a significant amount of the orthogonal solvent (anti‐solvent) just a few seconds before the end of the substrate rotation to induce rapid nucleation to produce nuclei covering the whole substrate. This process can be demonstrated successfully with the LaMer crystallization model.^[^ ^36^ ^]^ The subsequent crystal growth (will be affected by the distribution and size of the initial nuclei) is realized by thermal annealing of the film to yield a crystalline perovskite. Nevertheless, to prepare large‐area perovskite film, huge amount of orthogonal solvent need to be poured on the wet film rapidly and homogeneously, which is a very expensive, high pollution and difficult process. Therefore, for preparing large‐area perovskite film, anti‐solvent bathing instead of anti‐solvent dripping was generally used. Furthermore, it was known that ethyl ether acted as an agent for simultaneous evaporation of DMSO and DMF at similar temperatures to make dense perovskite film.^[^ ^37^ ^]^ Therefore, diethyl ether, instead of common used chlorobenzene, was used as an anti‐solvent for crystallizing the large‐area perovskite films with bathing method. The PCEs of the resulting PSMs were summarized in **Table** **1** (the corresponding *I–V* curves were displayed in Figure S2, Supporting Information). Data listed in Table 1 demonstrated that the mixture of GBL and 2P is the best solvent (among the four solvents used in this study) for the precursor solution to combine with diethyl ether anti‐solvent bathing for preparing large‐area perovskite film, however the PCEs of all PSMs are very low. Scanning electron microscopy (SEM) topographic images displayed in **Figure** **2** clearly reveal that low‐quality perovskite films with lots openings were formed when DMF or 2P was used as a solvent for perovskite precursor solution and diethyl ether as an anti‐solvent. Although continuous perovskite films can be made by using GBL or GBL:2P mixture as a solvent, the grains of the films are very small having a large amount of grain boundary defects, all result in low PCE of the corresponding PSMs. To improve the quality of perovskite film to enhance the photovoltaic performance of the solar modules, a new effective manipulation method for the crystallization process to control the scalable perovskite film deposition is required.

**Table 1:** The photovoltaic parameters of the PSMs based on the perovskite prepared from the precursor solution using different solvents and crystallization methods

Solvent for the precursor solution	*I*_sc_ [mA]	*V*_oc_ [V]	FF	PCE [%]
Perovskite film prepared with anti‐solvent bathing				
DMF	18.53	6.85	0.43	2.17
GBL	23.16	7.59	0.53	3.70
2P	25.68	7.95	0.56	4.54
GBL:2P = 4:1	33.49	8.01	0.59	6.28
Perovskite film prepared with freely falling anti‐solvent extraction				
*d* [m]	*I*_sc_ [mA]	*V*_oc_ [V]	FF	PCE [%]
0.2	38.35	8.30	0.64	8.08
0.8	42.95	8.53	0.68	9.89
2.2	44.77	8.65	0.72	11.06

**Figure 3:**
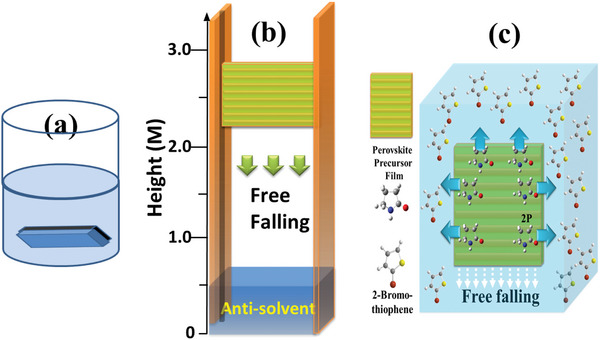
Schematic representation of the anti‐solvent extraction applied on large‐area perovskite precursor film. a) General anti‐solvent bathing. b) Freely falling anti‐solvent extraction. c) The extraction of the solvent (of the precursor solution) by anti‐solvent.

As mentioned previously, the preparation of perovskite film from its precursor solution involves solvent removal, nucleation, and crystal growth three stages. Nucleation and crystal growth strongly depend on the drying (solvent removal) kinetics of the perovskite wet film. As a result, choosing proper anti‐solvent and solvent removal method to find the balance between the three steps is of great importance for obtaining high‐quality large‐area perovskite films. Rapid solvent removal from the perovskite wet film to suppress the overgrowth of individual nuclei into large dendritic structures is a possible strategy. Vaynzof et al.^[^ ^38^ ^]^ had done the detailed structural, compositional, and microstructural characterization of perovskite films prepared by 14 anti‐solvents. They then identify two key factors that influence the quality of the resulting perovskite films: the miscibility between anti‐solvent and the solvent(s) of the perovskite precursor solution and the solubility of the perovskite precursors in the anti‐solvent, which combine to produce crystallization rate‐dependent behavior during the anti‐solvent application (dripping or bathing) step. Furthermore, to grow homogeneous large‐area crystalline perovskite film, the solvent of the precursor solution should also be extracted homogeneously by the anti‐solvent as fast as possible. In general, to crystalize the large‐area perovskite film by anti‐solvent bathing, the substrate (with perovskite precursor film) is dipped in the anti‐solvent bath with the substrate parallel to the bottom of the bath as illustrated in **Figure** **3**a. The solvent (of the perovskite precursor solution) extracted by the anti‐solvent stays closely to the perovskite film to obstruct the wet film contacting the fresh anti‐solvent to rapidly extract the rest precursor solvent. Therefore, low‐quality large‐area perovskite film was formed. Learning from the previous drying methods^[^ ^10^, ^11^, ^12^, ^13^, ^14^, ^15^, ^16^, ^17^, ^18^, ^19^, ^20^, ^21^, ^22^ ^]^ to remove the solvent quickly from the perovskite wet film, a new method call “freely falling anti‐solvent extraction, FFASE” operated in ambient atmosphere was developed in this study. We put the large‐area perovskite wet film on a holder with a track and let it falling freely to the anti‐solvent bath (as illustrated in Figure 3b) and then bathing for 10 min. By tuning the distance (*d* value) between the substrate edge and the liquid level, the contact time of the perovskite wet film with fresh anti‐solvent can be controlled. The advantage of the FFASE method is that the wet film contacts continuously with fresh anti‐solvent during dipping/crystallization process. Thus, the solvent (of the precursor solution) extracted by anti‐solvent will not wander up and down around the perovskite nuclei. Consequently, the solvent (for precursor solution) can be rapidly extracted away and the produced nuclei covered homogeneously on the whole substrate. It was found that the higher the distance between the substrate edge and liquid level, the better the PCE of the corresponding PSMs, see the data listed also in Table 1 (the corresponding *I–V* curves were shown in Figure S3, Supporting Information). Unfortunately, due to the simple setup made by us, the glass substrate breaks when the “*d* value” is larger than 2.2 m. FFASE method can improve the quality of perovskite film to increase the efficiency of the corresponding PSMs, nevertheless, the PCE is still much lower than the data reported in solar cell efficiency table version 58.^[^ ^24^ ^]^ For further improving the photovoltaic performance of PSMs, the next step is to tune the anti‐solvent.

**Figure 4:**
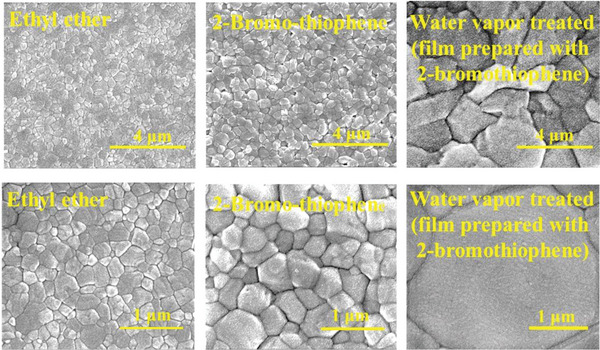
SEM images of perovskite films prepared using two different anti‐solvents and that prepared from 2‐bromothiophene anti‐solvent followed by water vapor annealing for 20 min.

In searching for more suitable anti‐solvents, 2‐bromothiophene was chosen due to several reasons: 1) Thiophene and its derivatives were well known as the units of semiconducting conjugated polymers, which consist of four *π* electrons from carbon atoms and two lone electron pairs from sulfur atom, enabling the electron‐rich conjugated *π* system^[^ ^39^ ^]^ with stable/reproducible redox reaction;^[^ ^40^ ^]^ 2) they are also usually used as sufficient hole extraction materials with the advantage of the highest occupied molecular orbital (HOMO) level and good hole mobility as well as hydrophobic nature to avoid attacking by water;^[^ ^41^, ^42^, ^43^, ^44^ ^]^ 3) the sulfur atom in thiophene may be able to passivate the perovskite defects by forming the bonding with the under‐coordinated Pb^2+^ in surface or at grain boundary;^[^ ^45^ ^]^ 4) bromine atom can also coordinate to the coordination unsaturated Pb^2+^ on the surface and the thiophene unit has better (than perovskite) compatibility to C_60_ (an ETL) via *π*–*π* interaction; 5) the miscibility between 2‐bromothiophene and 2P is better than that between ethyl ether and 2P, therefore 2‐bromothiophene can extract 2P from the perovskite precursor film quickly (as illustrated in Figure 3c); 6) the interaction between 2‐bromothiophene and perovskite precursor solution is stronger and 2‐bromothiophene has a higher viscosity, then moving (or leaving the perovskite film) more slowly (which suitable for growing bigger perovskite grains) compared to ethyl ether. In these aspects, 2‐bromothiophene can also act as a passivation agent as well as an anti‐solvent. When 2‐bromohiophene was used as an anti‐solvent combining with FFASE method, the highest PCE of the corresponding PSM increased to 12.35% with all photovoltaic parameters better than those of the PSM based on perovskite film fabricated by using ethyl ether as an anti‐solvent (see Figure S4, Supporting Information). The improvement in the photovoltaic performance of the PSM is due to the grain size of the perovskite film prepared using 2‐bromothiophene is larger than that of film prepared used ethyl ether anti‐solvent, as revealed by the SEM images displayed in **Figure** **4**. However, the grain size of the best photovoltaic performance film is much smaller than that of the small‐area perovskite film for the high‐efficiency PSCs we reported previously.^[^ ^46^, ^47^ ^]^ Therefore to enhance the PCE of the corresponding PSM, increasing the grain size of the large‐area film is the next effort.

It was believed that the fast increase in efficiency of PSCs is mainly from a large effort to reduce the defects of the perovskite absorber via tuning the perovskite compositions,^[^ ^48^ ^]^ advancing the film fabrication processes,^[^ ^8^ ^]^ smart interface engineering,^[^ ^49^, ^50^, ^51^ ^]^ defect passivation,^[^ ^52^, ^53^, ^54^ ^]^ etc. Grain boundaries are the typical defects of perovskite film, therefore the rather poor photovoltaic performance of the PSM may relate to the small grain size of the perovskite film. It was also shown that the cells based on perovskite absorber containing large multicrystalline grains have higher efficiency and better long‐term stability compared to those based on the film having small granules.^[^ ^55^, ^56^ ^]^ Therefore enlarging the grain size of perovskite film is an effective way to reduce the defects of perovskite to improve the photovoltaic performance of the corresponding PSMs. Post‐solvent annealing of perovskite film in a controlled environment has been demonstrated to be an efficient way to enlarge the grain size to enhance its PV performance.^[^ ^47^, ^57^ ^]^ We had also successfully used post treatment to enlarge the grains of perovskite film by treating the film with the chlorobenzene (anti‐solvent) containing small amount of H_2_O via spin‐coating.^[^ ^46^ ^]^ The function of the H_2_O was to dissolve the periphery of the perovskite grains followed by drying to mend the grain boundaries. This Ostwald ripening process can remove pinholes, heal the structural defects as well as increase the grain size and crystallinity of the perovskite film.^[^ ^46^ ^]^ Using the similar principle, we spun (1000 rpm) the large‐area perovskite film in a close system having the relative humidity of 60% for a period of time as illustrated in one of the steps shown in Figure 1. It was found that when the large‐area perovskite film was spun for 20 min at 60% relative humidity and then heated at 80 °C for 10 min, the corresponding PSMs achieve the highest PCE of 16.04% (the detailed photovoltaic parameters are listed in **Table** **2**, the photos of the PSMs were displayed in Figure S5, Supporting Information, and the related *I–V* curves were displayed in Figure S6, Supporting Information). By controlling the water vapor annealing time properly, the PCE of the corresponding PSM increases ≈30% of that for the PSM without post treating the perovskite absorber, mainly in *J*_sc_. The enhancement in PV performance is attributed reasonably to the quality of the perovskite film which was improved by post treating with water vapor (humidity). To analyze the cause of *J*_sc_ elevation, cells with small area (perovskite film diced from the 30 cm × 40 cm substrate) were demonstrated since only small area of the cell was used to take the EQE data. The *I–V* and EQE curves of the PSCs based on perovskite films prepared without post treatment and post water vapor annealing for 20 min were shown in Figure S7, Supporting Information. The EQE data reveal that after treating with water vapor the photo‐to‐current conversion efficiency of the corresponding cell increased at the whole visible light, suggesting better quality perovskite film was obtained by post water vapor annealing.

**Table 2:** Photovoltaic parameters of PSMs fabricated with various methods and measured at three temperatures

Condition	*I*_sc_ [mA]	*V*_oc_ [V]	FF	PCE [%]
without post treatment	48.89	8.73	0.74	12.53
Post treating the perovskite film with water vapor for various times				
10 min.	53.82	8.85	0.78	14.74
20 min.	56.01	9.02	0.80	16.04
30 min.	55.96	9.01	0.80	16.00
Thermal cycling for five times then measured at different temperature				
measured at 25 °C	55.68	9.02	0.80	15.94
measured at −20 °C	55.82	9.04	0.80	16.02
measured at 80 °C	55.60	9.00	0.80	15.89

**Figure 5:**
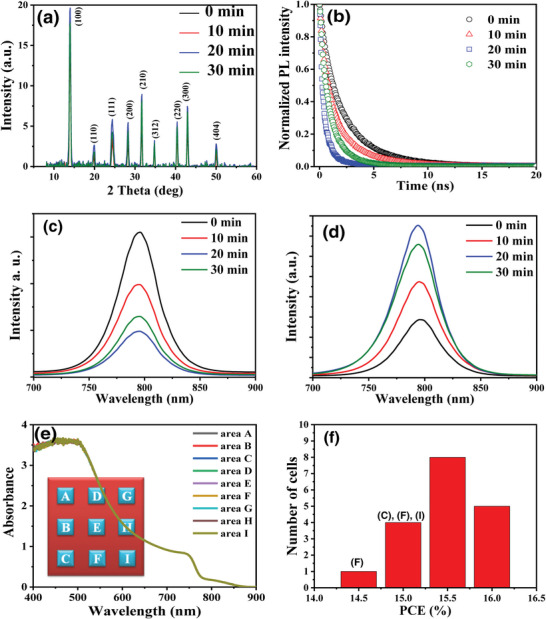
a) XRD patterns, b) time‐resolved photoluminescence curves of perovskite films deposited on LiCoO_2_ HTL, c) PL spectra of perovskite films deposited on LiCoO_2_ HTL, and d) PL spectra of perovskite films deposited on glass (without and with post water vapor annealing for different time). e,f) UV–vis spectra of the large‐area perovskite film at nine positions and the PCE histograms of the corresponding solar mini‐modules.

SEM topographies of the perovskite film after 20 min water vapor treatment are also displayed in Figure 4 (the SEM images and grain size statistics of perovskite film at different water vapor treating times are collected in Figure S8, Supporting Information). The SEM images clearly show that the grain size is significantly increased after post water vapor annealing: the largest size of 600 nm and over 3000 nm for the film before and after treating with water vapor for 20 min, then heated at 80 °C for 10 min, respectively. The grain size of water vapor treated perovskite film is even larger than that of film used to assemble 20% inverted PSC we reported previously.^[^ ^47^ ^]^ GIXRD patterns displayed in **Figure** **5**a revealed that both films (w/wo post water vapor annealing) have similar cell dimensions but the crystalline domains are smaller than the grain sizes which suggested that the films are all multicrystalline. Nevertheless, after post water vapor annealing not only the crystallinity but also the crystalline domain size of perovskite film enlarged (the detailed data were shown in Figure S9, Supporting Information), consistent with the grain size illustrated in Figure 4. Perovskite film having larger grain size and higher crystallinity facilitates the hole transport from the absorber to HTL as revealed with the time‐resolved photoluminescence (TRPL) curves (which were measured using an optical microscope system (Uni‐RAM, Protrustech) with the wavelength, pulse duration, and repetition rate of the excitation were 405 nm, 150 ps, and 20 MHz, respectively) displayed in Figure 5b. The charge recombination (average) lifetimes (*τ*_avg_) of the Psk excitons resolved by fitting the normalized TRPL curves are 2.18, 1.56, 0.46, and 0.99 ns for Psk films post treated with water vapor for 0, 10, 20, and 30 min, respectively. In a PSC the holes produced by exciting the absorber will diffuse quickly to the Psk/HTL interface and transferred to the HTL within several tens of nanoseconds. High‐quality perovskite film can transfer holes to HTL quickly to significantly reduce the radiative recombination within Psk film, therefore the PL quenches rapidly. The faster PL decay for Psk films post treated with water vapor (compared to that without post treatment) supported that post water vapor annealing can improve the quality of perovskite film to facilitate the hole transport and therefore enhance the photovoltaic performance of the corresponding PSMs. Consistent with the TRPL data, steady‐state PL of perovskite film deposited on LiCo_2_O_4_ HTL w/wo water vapor annealing were illustrated in Figure 5d. After post treatment of the perovskite film the PL intensity decreased suggesting that fast hole transport from excited perovskite film to HTL. On the other hand, when perovskite was deposited on inert glass substrate, film post treated with water vapor has higher luminescence intensity, suggesting the film has better quality compared to the film without post treatment. Furthermore, the thickness/quality of the large‐area perovskite film is very uniform, we measured the UV–vis absorption spectra at nine points of the film and the nine absorption curves almost overlap with each other (see Figure 5e). As a result, 10 × 10 mini‐modules (diced from the 30 × 40 module) have very close PCE as the histograms illustrated in Figure 5f. PSM based on the perovskite at lower position of the substrate (as indexed in Figure 5e) exhibited slightly lower efficiency. This is due to the wet film at low position contact the anti‐solvent with a fast speed, anti‐solvent was disturbed slightly, affecting the solvent extracting. Nevertheless, the effect in the position is not significant.

To resolve the passivation effect of 2‐bromothiophen, perovskite films with similar grains size were prepared by using 2‐bromothiophen and ethyl ether as anti‐solvents followed by post water vapor annealing for different times. The largest grain size of perovskite film can be obtained by using ethyl ether as an anti‐solvent. Therefore, perovskite film with smaller grain size (compared to the optimal film) was prepared to do the comparison. The photovoltaic performance of their corresponding PSMs was tested and the results were listed in Table S1, Supporting Information. The SEM images of the perovskite films were also displayed in Table S1, Supporting Information for easy comparison. Even the films have a similar grain size, perovskite prepared using 2‐bromothiophene anti‐solvent has better performance compared to that prepared from ethyl ether anti‐solvent, the passivation effect of 2‐bromothiophene was revealed. Furthermore, very weak sulfur signal on the XPS spectrum (Figure S10, Supporting Information) was detected from the perovskite film prepared from 2‐bromothiophene. XPS further supported that 2‐bromothiophene can passivate the perovskite film.

**Figure 6:**
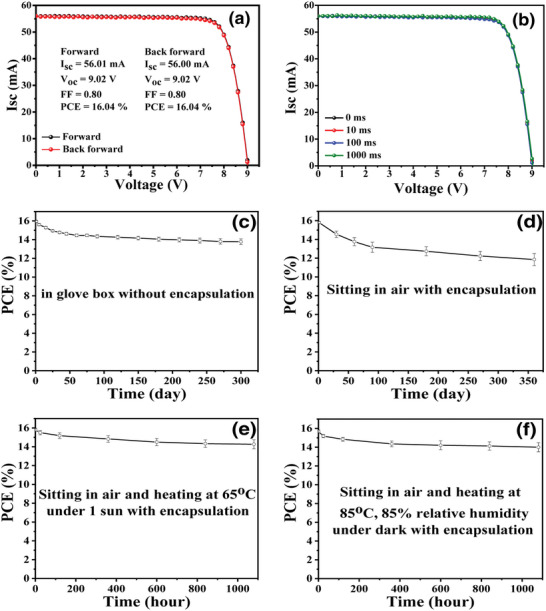
The stability of the high‐efficiency perovskite mini‐module (with error bars). a) *I*–*V* curves scanned at two directions. b) Scanned at different rates. c) Storing in glove box for 300 d without encapsulation. d) Sitting outside for 360 d with encapsulation. e) Heating at 65 °C under 1 sun for 1080 h (encapsulated PMS). e) Heating at 85 °C, 85% relative humidity under dark (encapsulated PMS).

It is notable that the high‐efficiency PSM achieves a remarkable high FF of 0.80 which is very close to the high‐efficiency perovskite module reported very recently.^[^ ^58^, ^59^ ^]^ Therefore, we believe that the lower efficiency of our PSM (compared to the literature reports^[^ ^58^, ^59^ ^]^) may not be due to the quality of perovskite film, most probably because of the imperfect electric patterns of our modules which were made manually. To confirm this speculation, the cells with active area of 0.1 and 2.8 cm^2^ (diced from the 30 cm × 40 cm substrate) achieve the high efficiency of 22.15% and 20.89%, respectively, proved that perovskite film fabricated by the method described in this paper has very high quality. The *I–V* curves of the small‐area cells were displayed in Figure S7a,d, Supporting Information. The PSMs have almost no current hysteresis, the PCE is independent of voltage scan rates and scan directions as the *I–V* curves shown in **Figure** **6**a,b. The results suggested that the large‐area perovskite film has small amount of defects and very balanced in electron and hole transport.^[^ ^60^ ^]^ The PSMs are also very stable. Without packing, PSM lost 12% by storing in glove box for 300 d under room lighting (Figure 6c). With proper packing, the PSM kept 80% of its original efficiency after sitting in an ambient atmosphere (25 °C, average 50% related humidity controlled with air conditioning) for 360 d (Figure 6d). The stability of the encapsulated PSMs under stressed conditions, such as at 60 °C under AM1.5 (100 mW cm^−2^) illumination for 1000 h or heating at 85 °C, 85% relative humidity for 1000 h under dark, were also tested and the PSMs can keep 92% of its original efficiency (Figure 6e,f).

Perovskite solar cells are promising for terrestrial energy production, which opens new opportunities for low‐cost space power, due to their high specific power and appropriate spectrum response.^[^ ^61^ ^]^ A comprehensive analysis^[^ ^62^ ^]^ also pointed out a massive cost reduction of $10–$20 billion USD if perovskite PV (instead of the conventional GaAs PV) is used in the expanding low earth orbit (LEO) satellites market. However, delivering on these promises will require overcoming lots of environmental challenges associated with the space environment. Those challenges include the stability under thermal cycling, radiation (ultraviolet and atomic oxygen) tolerance, micrometeoroids damages, etc.^[^ ^63^ ^]^ The severe change in temperature and light intensity associated with space environment was known to be two of the big challenges for production sufficient electricity in satellites. We test the stability of PSM under thermal cycling between −20 and 80 °C in vacuum (6.5 × 10^−3^ Pa) and measure the photovoltaic performance of PSM at temperature at −20 and 80 °C. The resulting photovoltaic parameters are also listed in Table 2 (the corresponding *I–V* curves as well as the record of thermal cycling are collected in Figure S11, Supporting Information). The PCEs of the PSM change very slightly (less than 0.10% absolutely) after five times of the thermal cycling and the efficiencies measured at the temperatures of −20 to 80 °C are similar to that evaluated at room temperature (25 °C). The results suggested that PSMs have a great potential to be used as a space power.

Finally, high efficiency under indoor lighting was known as one of the special advantages of the new (third) generation PVs. The attraction for the development of efficient solar cells under indoor lighting is because of the Internet of Things (IoTs) revolution. The use of indoor PVs in a large amount of self‐powered, easy‐to‐install wireless devices for an IoT or building management systems can reduce the use of batteries and battery replacement, saving both energy and manpower.^[^ ^64^ ^]^ Indoor (or called artificial) light is usually defined as 1000 lux fluorescent or light‐emitting diodes (LEDs) light^[^ ^65^, ^66^ ^]^ and the luminance levels of indoor light for measuring the PV performance suggested by Reese et al.^[^ ^67^ ^]^ are 200 lux (living room‐like), 500 lux (office‐like), and 1000 lux (supermarket‐like). Indoor light has a power density thousands times lower than 1 sun standard light (AM1.5G, 100 mW cm^−2^). The low incident power of indoor light generates only very small amount of electrons (and holes), therefore the trap (or defect) density becomes critical due to a high ratio of trapped electrons to photogenerated electrons. In other words, the efficiency of the cell/module under indoor lighting is more affected by the density of defects or trap sites (therefore the quality) of the perovskite film than those under standard 1sun illumination. PSCs have demonstrated themselves as an efficient weak‐light energy harvester with the PCE of 24%–40% under the indoor light intensity of 400–1000 lux in several studies.^[^ ^66^, ^68^, ^69^, ^70^, ^71^, ^72^ ^]^ However, those data are based on the small cells with a typical size of 0.1–5 cm^2^. To achieve a high‐efficiency module, the entire large‐area film is required to be almost defect free. Even a single pinhole may cause the shunt of a subcell, which dramatically reduces the photovoltaic performance of the module under weak lighting. Therefore, to our best knowledge, only one paper reported by Watson and co‐workers^[^ ^73^ ^]^ was found regarding the detailed photovoltaic performance of PSM under indoor lighting. They fabricated an all‐printable large‐area perovskite solar submodule with an active area of 198 cm^2^ on an A4‐sized glass substrate (aperture area: 435.6 cm^2^) to achieve 6.6% efficiency under 1‐sun illumination. Under the fluorescent lamp, this submodule produces 2, 6, and 10 mW power under 200, 600, and 1000 lux illumination, which corresponds to ≈10, 30, and 50 µW cm^−2^ power, respectively. To demonstrate the good quality perovskite film fabricated via FFASE followed by water vapor annealing, the performance of the PSM under T5 fluorescent lamp (the spectral irradiance of T5 lamp used in this study was displayed in Figure S12a, Supporting Information) at three different intensities was test. At the same time a crystalline silicon solar module with similar active area (36 cm^2^) and efficiency (16.34% under 1 sun, 100 mW cm^−2^) was also measured simultaneously to be a reference and the data were summarized in **Table** **3** (all *I–V* curves were displayed in Figure S12, Supporting Information). The performance of the crystalline silicon mini‐module under indoor lighting is close to the polycrystalline silicon mini‐module (active area of 68.64 cm^2^) produced by Multicomp‐Pro,^[^ ^74^ ^]^ indicated that the indoor light measuring setup can be trusted. Our mini‐module shows excellent efficiency and voltage response even under extremely low illumination of 298 lux. The efficiency of 87, 47, and 21 µW cm^−2^ were obtained at fluorescent light intensity of 945, 537, and 298 lux, respectively. As mentioned previously, a single pinhole may reduce the performance of the mini‐module under indoor light. Therefore to achieve a high‐efficiency module, the entire large‐area film should be almost defect free. Our mini‐module has high efficiency under indoor lighting demonstrated that the large‐area perovskite film has very few defects. It is no easy feat considering that translating to large‐area film often results in increased defects and sheet resistance of perovskite film, adversely affecting the module performance. The high PV performance under indoor light of PSM suggested that the new creative FFASE and water vapor annealing methods reported in this paper are suitable to fabricate high‐quality, large‐area perovskite films.

**Table 3:** Photovoltaic parameters of PSM and crystalline silicon mini‐module under indoor light

Light intensity	*I*_sc_ [mA]	*V*_oc_ [V]	FF	PCE [%]	*P*_max_
Perovskite solar mini‐module with active area of 25.2 cm^2^ (nine cells)					
945 lux	0.339	7.987	0.778	30.76	2.11
537 lux	0.192	7.792	0.758	29.07	1.14
298 lux	0.111	7.627	0.736	28.58	0.524
Crystalline silicon solar mini‐module with active area of 36 cm^2^					
945 lux	1.50	0.55	0.44	3.55	0.36
537 lux	0.84	0.45	0.42	2.70	0.16
298 lux	0.46	0.35	0.39	2.00	0.06

## 3 Conclusion

In conclusion, high‐quality, large‐area perovskite film on a 30 cm × 40 cm ITO substrate prepared by spin‐coating, freely falling anti‐solvent extraction, and post water vapor annealing under an ambient atmosphere was demonstrated. The resulting diced 10 cm × 10 cm perovskite mini‐module (with a mask active area of 25.2 cm^2^) achieves the highest PCE of 16.04% under 1 sun, 100 mW cm^−2^ (at temperature range from −20 to 80 °C) with negligible current hysteresis. The mini‐module has also very high PCE of 30% under 945 lux T5 light and very stable under thermal cycling which may also able to applied in low earth orbit satellites. The perovskite film fabrication process reported in this paper provides an appealing method for fabricating large‐area, high‐quality perovskite films reproducibly for scalable perovskite module with long‐term stability. This study was able to prepare high‐quality perovskite film with easy reproducible way to achieve high efficiency devices via solution process making PSMs to be competitive with the existing photovoltaic devices.

## Conflict of Interest

The authors declare no conflict of interest.

## Author Contributions

C.‐G.W. conceived and directed the overall project as well as supervised the research and wrote the manuscript. C.‐H.C. fabricated all the devices and conducted all characterization and data analysis. Both authors read and commented on the manuscript.

## Supporting information

Supporting information

## Data Availability

The data that supports the findings of this study are available in the supplementary material of this article.

## References

[1] N. Li , X. Niu , Q. Chen , H. Zhou , Chem. Soc. Rev. 2020, 49, 8235. 32909584 10.1039/d0cs00573h

[2] N. S. Kumar , C. B. Naidu , J. Materiomics 2021, 7, 940.

[3] P. Zhang , M. Li , W.‐C. Chen , Front. Chem. 2022, 10, 802890. 35480386 10.3389/fchem.2022.802890 PMC9035841

[4] M. Kim , J. Jeong , H. Lu , T. K. Lee , F. T. Eickemeyer , Y. Liu , I. Choi , S. Choi , Y. Jo , H. Kim , S. Mo , Y. Kim , H. Lee , N. An , S. Cho , W. R. Tress , S. M. Zakeeruddin , A. Hagfeldt , J. Kim , M. Grätzel , D. Kim , Science 2022, 375, 302. 35050659 10.1126/science.abh1885

[5] Z. Li , B. Li , X. Wu , S. A. Sheppard , S. Zhang , D. Gao , N. J. Long , Z. Zhu , Science 2022, 376, 416. 35446656 10.1126/science.abm8566

[6] National Renewable Energy Laboratory, Best Research Cell Efficiency, http://www.nrel.gov/ncpv/images/efficiency_chart.jpg (accessed: July 2022).

[7] F. Matteocci , S. Razza , F. D. Giacomo , S. Casaluci , G. Mincuzzi , T. M. Brown , A. D'Epifanio , S. Licoccia , A. D. Carlo , Phys. Chem. Chem. Phys. 2014, 16, 3918. 24452004 10.1039/c3cp55313b

[8] N. J. Jeon , J. H. Noh , Y. C. Kim , W. S. Yang , S. Ryu , S. I. Seok , Nat. Mater. 2014, 13, 897. 24997740 10.1038/nmat4014

[9] M. Xiao , F. Huang , W. Huang , Y. Dkhissi , Y. Zhu , J. Etheridge , A. Gray‐Weale , U. Bach , Y. B. Cheng , L. Spiccia , Angew. Chem., Int. Ed. 2014, 126, 10056. 10.1002/anie.201405334 25047967

[10] Y. Zhou , M. Yang , W. Wu , A. L. Vasiliev , K. Zhu , N. P. Padture , J. Mater. Chem. A 2015, 3, 8178.

[11] M. Yang , Y. Zhou , Y. Zeng , C. S. Jiang , N. P. Padture , K. Zhu , Adv. Mater. 2015, 27, 6363. 26414514 10.1002/adma.201502586

[12] W. Nie , H. Tsai , R. Asadpour , J.‐C. Blancon , A. J. Neukirch , G. Gupta , J. J. Crochet , M. Chhowalla , S. Tretiak , M. A. Alam , Science 2015, 347, 522. 25635093 10.1126/science.aaa0472

[13] A. T. Mallajosyula , K. Fernando , S. Bhatt , A. Singh , B. W. Alphenaar , J.‐C. Blancon , W. Nie , G. Gupta , A. D. Mohite , Appl. Mater. Today 2016, 3, 96.

[14] Y. Deng , E. Peng , Y. Shao , Z. Xiao , Q. Dong , J. Huang , Energy Environ. Sci. 2015, 8, 1544.

[15] S. Tang , Y. Deng , X. Zheng , Y. Bai , Y. Fang , Q. Dong , H. Wei , J. Huang , Adv. Energy Mater. 2017, 7, 1700302.

[16] F. Huang , Y. Dkhissi , W. Huang , M. Xiao , I. Benesperi , S. Rubanov , Y. Zhu , X. Lin , L. Jiang , Y. Zhou , Nano Energy 2014, 10, 10.

[17] K. Hwang , Y.‐S. Jung , Y.‐J. Heo , F. H. Scholes , S. E. Watkins , J. Subbiah , D. J. Jones , D.‐Y. Kim , D. Vak , Adv. Mater. 2015, 27, 1241. 25581092 10.1002/adma.201404598

[18] Q. Hu , L. C. Zhao , J. Wu , K. Gao , D. Y. Luo , Y. F. Jiang , Z. Y. Zhang , C. H. Zhu , E. Schaible , A. Hexemer , C. Wang , Y. Liu , W. Zhang , M. Gratzel , F. Liu , T. P. Russell , R. Zhu , Q. H. Gong , Nat. Commun. 2017, 8, 15688. 28635947 10.1038/ncomms15688 PMC5482054

[19] L.‐L. Gao , C.‐X. Li , C.‐J. Li , G.‐J. Yang , J. Mater. Chem. A 2017, 5, 1548.

[20] X. Li , D. Bi , C. Yi , J.‐D. Décoppet , J. Luo , S. M. Zakeeruddin , A. Hagfeldt , M. Grätzel , Science 2016, 353, 58. 27284168 10.1126/science.aaf8060

[21] L.‐L. Gao , L.‐S. Liang , X.‐X. Song , B. Ding , G.‐J. Yang , B. Fan , C.‐X. Li , C.‐J. Li , J. Mater. Chem. A 2016, 4, 3704.

[22] B. Ding , L. Gao , L. Liang , Q. Chu , X. Song , Y. Li , G. Yang , B. Fan , M. Wang , C. Li , ACS Appl. Mater. Interfaces 2016, 8, 20067. 27428311 10.1021/acsami.6b05862

[23] M. A. Green , E. D. Dunlop , J. Hohl‐Ebinger , M. Yoshita , N. Kopidakis , K. Bothe , D. Hinken , M. Rauer , X. Hao , Prog. Photovoltaics: Res. Appl. 2022, 30, 687.

[24] M. A. Green , E. D. Dunlop , J. Hohl‐Ebinger , M. Yoshita , N. Kopidakis , K. Bothe , D. Hinken , M. Rauer , X. Hao , Prog. Photovolt. Res. Appl. 2021, 29, 657.

[25] F. Guo , S. Qiu , J. Hu , H. Wang , B. Cai , J. Li , X. Yuan , X. Liu , K. Forberich , C. J. Brabec , Y. Mai , Adv. Sci. 2019, 6, 1901067. 10.1002/advs.201901067 PMC6724353 31508290

[26] J. M. Ball , M. M. Lee , A. Hey , H. Snaith , Energy Environ. Sci. 2013, 6, 1739.

[27] M. J. Carnie , C. Charbonneau , M. L. Davies , J. Troughton , T. M. Watson , K. Wojciechowski , H. Snaith , D. A. Worsley , Chem. Commun. 2013, 49, 7893. 10.1039/c3cc44177f 23900427

[28] G. E. Eperon , V. M. Burlakov , P. Docampo , A. Goriely , H. J. Snaith , Adv. Funct. Mater. 2014, 24, 151.

[29] Q. Wang , Y. C. Shao , Q. F. Dong , Z. Q. Xiao , Y. B. Yuan , J. S. Huang , Energy Environ. Sci. 2014, 7, 2359.

[30] C.‐H. Chiang , C.‐C. Chen , K. M. Nazeeruddine , C.‐G. Wu , J. Mater. Chem. A 2018, 6, 13751.

[31] Z. P. Wang , D. P. McMeekin , N. Sakai , S. van Reenen , K. Wojciechowski , J. B. Patel , M. B. Johnston , H. J. Snaith , Adv. Mater. 2017, 29, 1604186. 10.1002/adma.201604186 27905138

[32] L. Zeng , S. Chen , K. Forberich , C. J. Brabec , Y. Mai , F. Guo , Energy Environ. Sci. 2020, 13, 4666.

[33] N. Ahn , D. Y. Son , I. H. Jang , S. M. Kang , M. Choi , N. G. Park , J. Am. Chem. Soc. 2015, 137, 8696. 26125203 10.1021/jacs.5b04930

[34] C.‐H. Chiang , Z.‐L. Tseng , C.‐G. Wu , Sol. RRL 2020, 4, 1900402.

[35] M. Xiao , F. Huang , W. Huang , Y. Dkhissi , Y. Zhu , J. Etheridge , A. Gray‐Weale , U. Bach , Y. B. Cheng , L. A. Spiccia , Angew. Chem., Int. Ed. Engl. 2014, 53, 9898. 25047967 10.1002/anie.201405334

[36] C. B. Whitehead , S. Özkar , R. G. Finke , Chem. Mater. 2019, 31, 7116.

[37] K.‐H. Hwang , S. H. Nam , D. I. Kim , H. J. Seo , J.‐H. Boo , Sol. Energy Mater. Sol. Cells 2018, 180, 386.

[38] A. D. Taylor , Q. Sun , K. P. Goetz , Q. An , T. Schramm , Y. Hofstetter , M. Litterst , F. Paulus , Y. Vaynzof , Nat. Commun. 2021, 12, 1878. 33767163 10.1038/s41467-021-22049-8 PMC7994557

[39] C. Edmiston , K. Ruedenberg , Rev. Mod. Phys. 1963, 35, 457.

[40] C.‐G. Wu , J. Chin. Chem. Soc. 2022, 69, 1242.

[41] E. Edri , S. Kirmayer , D. Cahen , G. Hodes , J. Phys. Chem. Lett. 2013, 4, 897. 26291353 10.1021/jz400348q

[42] W. Yan , Y. Li , Y. Li , S. Ye , Z. Liu , S. Wang , Z. Bian , C. Huang , Nano Res. 2015, 8, 2474.

[43] C.‐Y. Chang , H.‐H. Huang , H. Tsai , S.‐L. Lin , P.‐H. Liu , W. Chen , F.‐C. Hsu , W. Nie , Y.‐F. Chen , L. Wang , Adv. Sci. 2021, 8, 2002718. 10.1002/advs.202002718 PMC7927620 33717841

[44] T. Y. Wen , S. Yang , P. F. Liu , L. J. Tang , H. W. Qiao , X. Chen , X. H. Yang , Y. Hou , H. G. Yang , Adv. Energy Mater. 2018, 8, 1703143.

[45] T. H. Wu , Y. B. Wang , X. Li , Y. Z. Wu , X. Y. Meng , D. Y. Cui , X. D. Yang , L. Y. Han , Adv. Energy Mater. 2019, 9, 1803766.

[46] C.‐G. Wu , C.‐H. Chiang , ACS Nano 2018, 12, 10355. 30280561 10.1021/acsnano.8b05731

[47] C.‐H. Chiang , K. M. Nazeeruddin , M. Grätzel , C.‐G. Wu , Energy Environ. Sci. 2017, 10, 808.

[48] N. J. Jeon , J. H. Noh , W. S. Yang , Y. C. Kim , S. Ryu , J. Seo , S. I. Seok , Nature 2015, 517, 476. 25561177 10.1038/nature14133

[49] M. Graetzel , R. A. J. Janssen , D. B. Mitzi , E. H. Sargent , Nature 2012, 488, 304. 22895335 10.1038/nature11476

[50] C. Sun , Z. H. Wu , H. L. Yip , H. Zhang , X. F. Jiang , Q. F. Xue , Z. C. Hu , Z. H. Hu , Y. Shen , M. K. Wang , F. Huang , Y. Cao , Adv. Energy Mater. 2016, 6, 1501534.

[51] J. L. Hu , J. You , C. Peng , S. D. Qiu , W. X. He , C. H. Li , X. H. Liu , Y. H. Mai , F. Guo , Sol. RRL 2020, 4, 1900384.

[52] Q. Jiang , Y. Zhao , X. W. Zhang , X. L. Yang , Y. Chen , Z. M. Chu , Q. F. Ye , X. X. Li , Z. G. Yin , J. B. You , Nat. Photonics 2019, 13, 460.

[53] F. Gao , Y. Zhao , X. W. Zhang , J. B. You , Adv. Energy Mater. 2020, 10, 1902650.

[54] B. Chen , P. N. Rudd , S. Yang , Y. B. Yuan , J. S. Huang , Chem. Soc. Rev. 2019, 48, 3842. 31187791 10.1039/c8cs00853a

[55] G. E. Eperon , V. M. Burlakov , P. Docampo , A. Goriely , H. J. Snaith , Adv. Funct. Mater. 2014, 24, 151.

[56] C.‐H. Chiang , C.‐G. Wu , ChemSusChem 2016, 9, 2666. 27601006 10.1002/cssc.201600887

[57] C. Liu , K. Wang , C. Yi , X. J. Shi , A. W. Smith , X. Gong , A. J. Heeger , Adv. Funct. Mater. 2016, 26, 101.

[58] S. Chen , X. Dai , S. Xu , H. Jiao , L. Zhao , J. Huang , Science 2021, 373, 902. 34413234 10.1126/science.abi6323

[59] K. Xiao , Y.‐H. Lin , M. Zhang , R. D. J. Oliver , X. Wang , Z. Liu , X. Luo , J. Li , D. Lai , H. Luo , R. Lin , J. Xu , Y. Hou , H. J. Snaith , H. Tan , Science 2022, 376, 762. 35549402 10.1126/science.abn7696

[60] H. J. Snaith , A. Abate , J. M. Ball , G. E. Eperon , T. Leijtens , N. K. Noel , S. D. Stranks , J. T.‐W. Wang , K. Wojciechowski , W. Zhang , J. Phys. Chem. Lett. 2014, 5, 1511. 26270088 10.1021/jz500113x

[61] M. Kaltenbrunner , G. Adam , E. D. Głowacki , M. Drack , R. Schwödiauer , L. Leonat , D. H. Apaydin , H. Groiss , M. C. Scharber , M. S. White , Nat. Mater. 2015, 14, 1032. 26301766 10.1038/nmat4388

[62] A. W. Y. Ho‐Baillie , H. G. J. Sullivan , T. A. Bannerman , H. P. Talathi , J. Bing , S. Tang , A. Xu , D. Bhattacharyya , I. H. Cairns , D. R. McKenzie , Adv. Mater. Technol. 2022, 7, 2101059.

[63] A. R. Kirmani , B. K. Durant , J. Grandidier , N. M. Haegel , M.i. D. Kelzenberg , Y. M. Lao , M. D. McGehee , L. McMillon‐Brown , D. P. Ostrowski , T. J. Peshek , B. Rout , I. R. Sellers , M. Steger , D. Walker , D. M. Wilt , K. T. VanSant , J. M. Luther , Joule 2022, 6, 1015.

[64] N. Reich , W. Van Sark , W. Turkenburg , Renewable Energy 2011, 36, 642.

[65] X. Yue , M. Kauer , M. Bellanger , O. Beard , M. Brownlow , D. Gibson , C. Clark , C. MacGregor , S. Song , IEEE Internet Things J. 2017, 4, 2092.

[66] C. Y. Chen , J. H. Chang , K. M. Chiang , H. L. Lin , S. Y. Hsiao , H. W. Lin , Adv. Funct. Mater. 2015, 25, 7064.

[67] M. O. Reese , S. A. Gevorgyan , M. Jørgensen , E. Bundgaard , S. R. Kurtz , D. S. Ginley , D. C. Olson , M. T. Lloyd , P. Morvillo , E. A. Katz , A. Elschner , O. Haillant , T. R. Currier , V. Shrotriya , M. Hermenau , M. Riede , K. R. Kirov , G. Trimmel , T. Rath , O. Ingan€as , F. Zhang , M. Andersson , K. Tvingstedt , M. Lira‐Cantu , D. Laird , C. McGuiness , S. (Jimmy) Gowrisanker , M. Pannone , M. Xiao , J. Hauch , et al., Sol. Energy Mater. Sol. Cells 2011, 95, 1253e1267.

[68] C.‐Y. Chen , W.‐H. Lee , S.‐Y. Hsiao , W.‐L. Tsai , L. Yang , H.‐L. Lin , H.‐J. Chou , H.‐W. Lin , J. Mater. Chem. A 2019, 7, 3612.

[69] M. Li , C. Zhao , Z.‐K. Wang , C.‐C. Zhang , H. K. H. Lee , A. Pockett , J. Barbé , W. C. Tsoi , Y.‐G. Yang , M. J. Carnie , Adv. Energy Mater. 2018, 8, 1801509.

[70] G. Lucarelli , F. di Giacomo , V. Zardetto , M. Creatore , T. M. Brown , Nano Res. 2017, 10, 2130.

[71] J. Dagar , S. Castro‐Hermosa , G. Lucarelli , F. Cacialli , T. M. Brown , Nano Energy 2018, 49, 290.

[72] C. Dong , X.‐M. Li , C. Ma , W.‐F. Yang , J.‐J. Cao , F. Igbari , Z.‐K. Wang , L.‐S. Liao , Adv. Funct. Mater. 2021, 31, 2011242.

[73] F. De Rossi , J. A. Baker , D. Beynon , K. E. A. Hooper , S. M. P. Meroni , D. Williams , Z. Wei , A. Yasin , C. Charbonneau , E. H. Jewell , T. M. Watson , Adv. Mater. Technol. 2018, 3, 1800156.

[74] Y. Li , N. J. Grabham , S. P. Beeby , M. J. Tudor , Sol. Energy 2015, 111, 21.

