# Supplementary material for: Large‐Area Perovskite Film Prepared by New FFASE Method for Stable Solar Modules Having High Efficiency under Both Outdoor and Indoor Light Harvesting
Source: Adv Sci (Weinh). 2023 Jan 16;10(7):2205967. doi: 10.1002/advs.202205967 (PMC9982567; doi:10.1002/advs.202205967)
Supplement: Supplementary file 1 — Supporting information [file ADVS-10-2205967-s001.pdf]

Electronic Supporting Information

Large-area perovskite film prepared by new FFASE method for stable solar modules having high-efficiency under both outdoor and indoor light harvesting

Chien-Hung Chiang[b,*], Chun-Guey Wu[a,b,*]

[a,] Department of Chemistry National Central University, Jhong-Li, Taiwan 32001, ROC.

[b] Research Center for New Generation Photovoltaics, National Central University, Jhong-Li, Taiwan 32001, ROC.

E-mail address of Professor C. G. Wu: t610002@cc.ncu.edu.tw

Table S1: The photovoltaic parameters of the PSMs based on perovskite films with similar grain size prepared with ethyl ether and 2-bromothiophene anti-solvents followed by post water vapor annealing for various time.

**Anti-solvent**	**Water vapor annealing time**	**Isc (mA)**	**Voc (V)**	**FF**	**PCE (%)**
Ethyl ether	20 min	52.34	8.75	0.74	13.45
2-bromothiophene	10 min	53.82	8.85	0.78	14.74

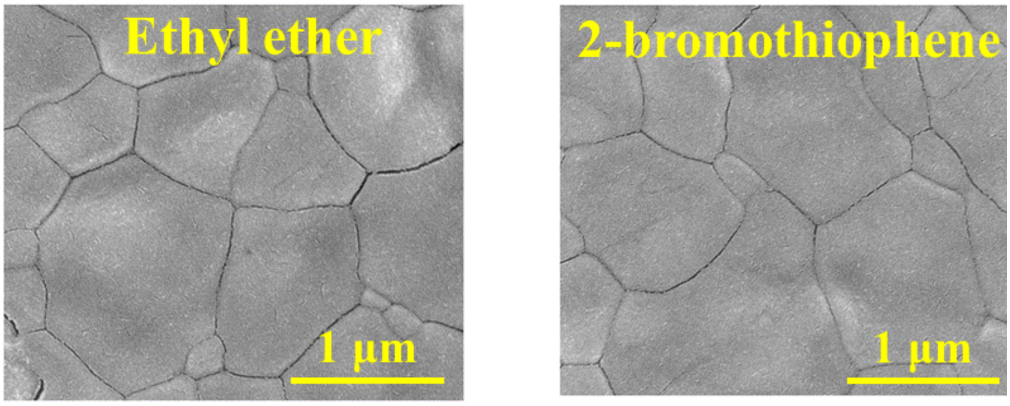

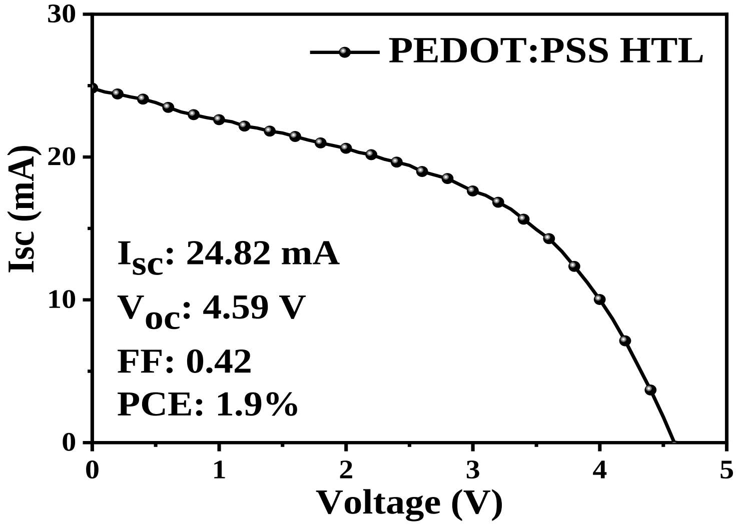

Figure S1: I-V curve of the PSM based on PEDOT:PSS HTL

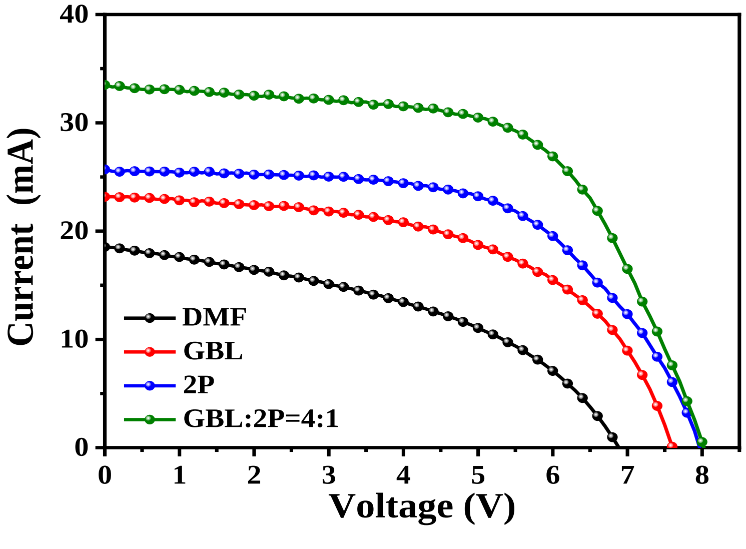

Figure S2: I-V curves of the PSMs based on perovskite film prepared form different solvents of the precursor solutions.

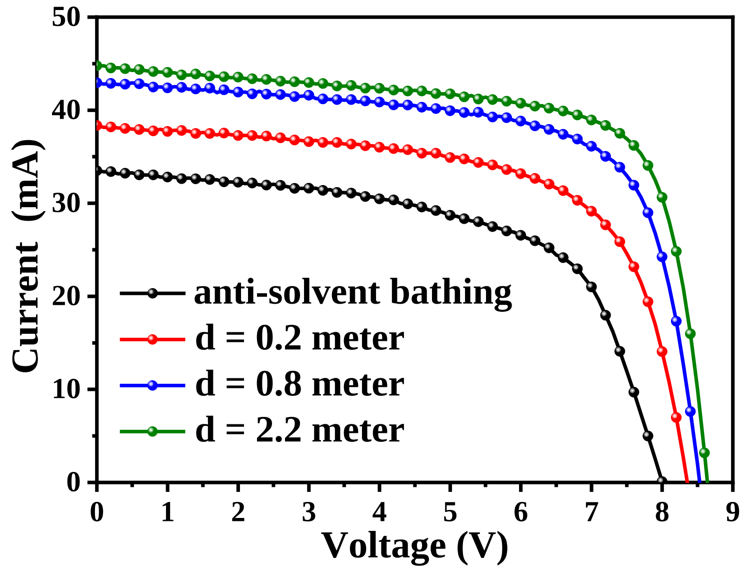

Figure S3: I-V curves of PSMs based on perovskite films prepared with various conditions.

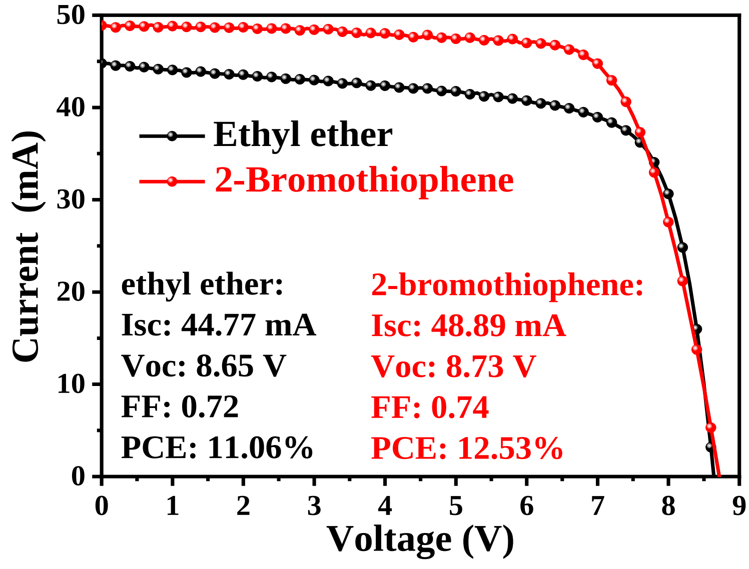

Figure S4: I-V curves and photovoltaic parameters of the PSMs based on perovskite film prepared with two anti-solvents.

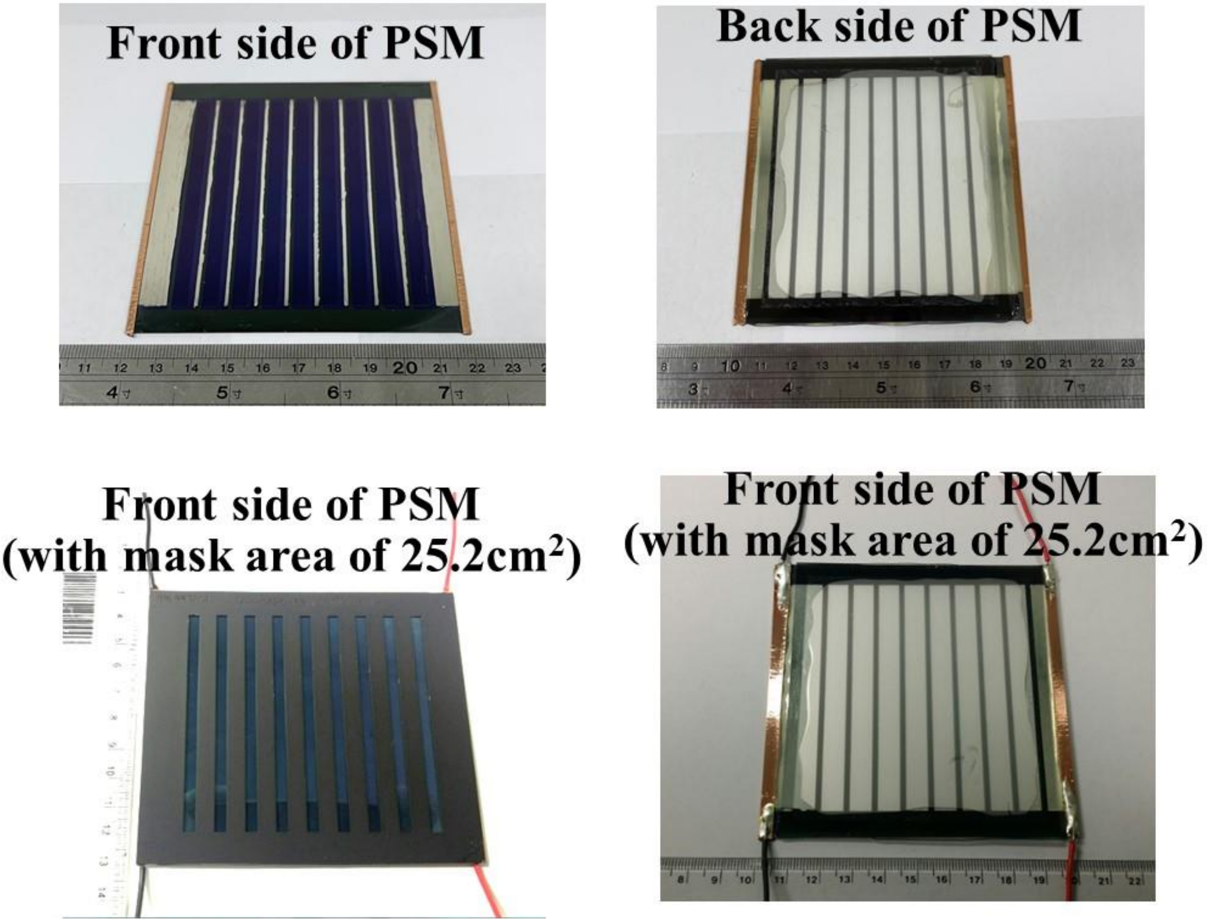

Figure S5: The photos of the perovskite solar mini-modules (PSMs)

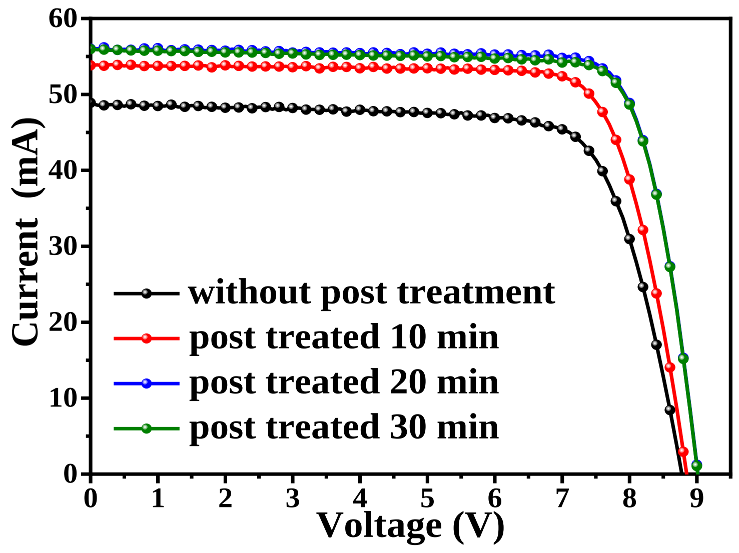

Figure S6: I-V curves of the PSMs based on perovskite film prepared without or with water vapor post treatment.

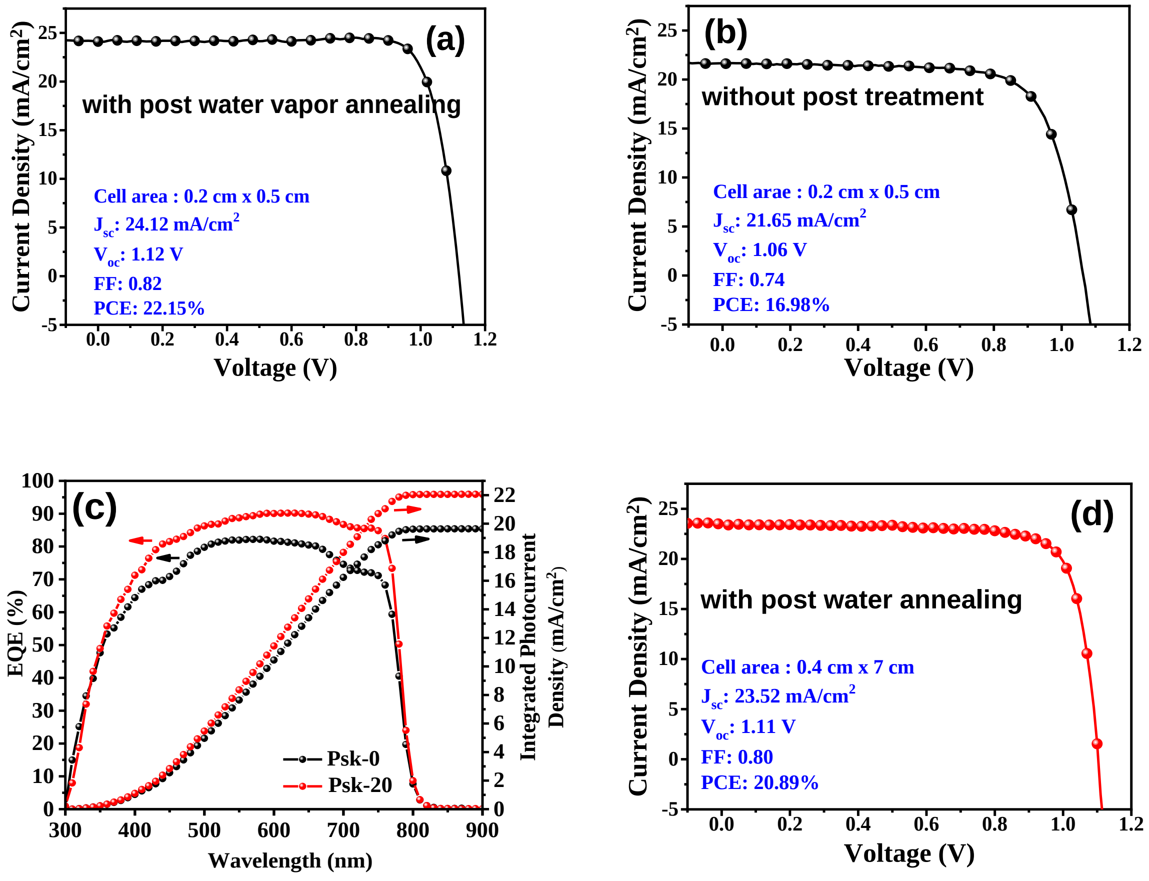

Figure S7: (a), (b) I-V curves with photovoltaic parameters of the small cell based on perovskite films w/wo water vapor annealing (c) the corresponding EQE curves of the small cell (d) I-V curves with photovoltaic parameters of the 2.8 cm^2 cell based on perovskite film treated by water vapor for 20 min.

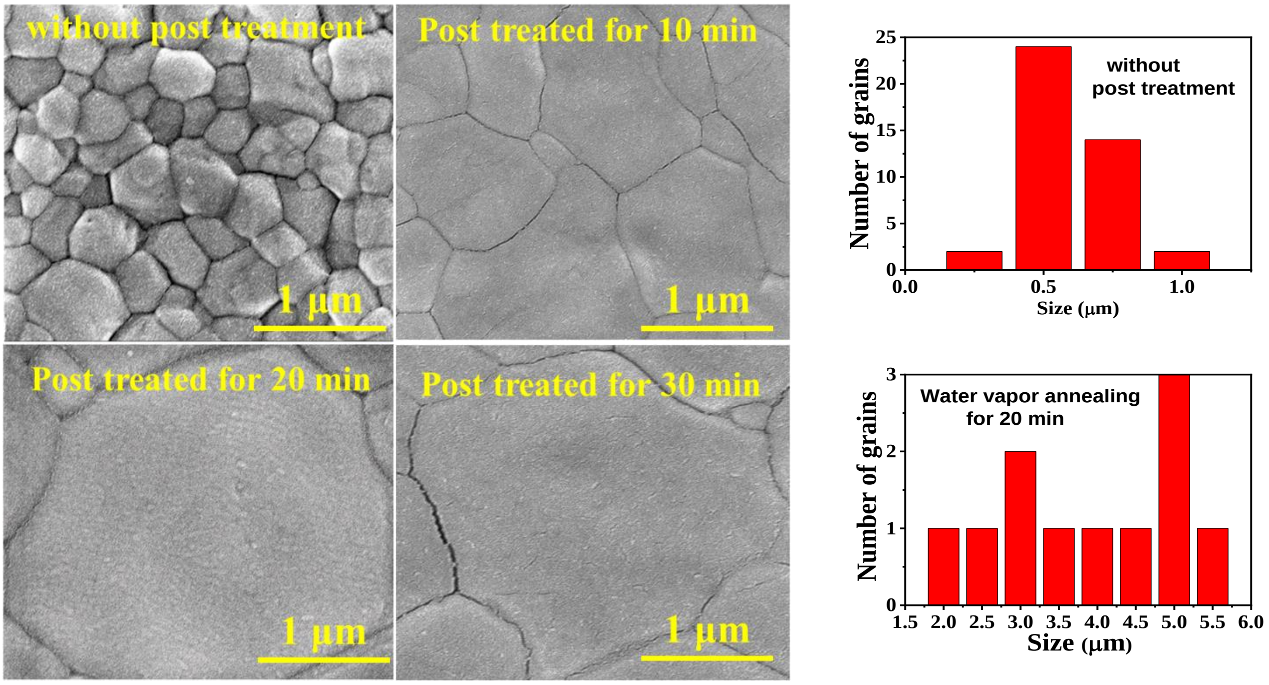

Figure S8: (left) SEM topographies of perovskite film prepared without or with water vapor post treatment. (right) the grain size statistics (estimated from the SEM images) of the perovskite films.

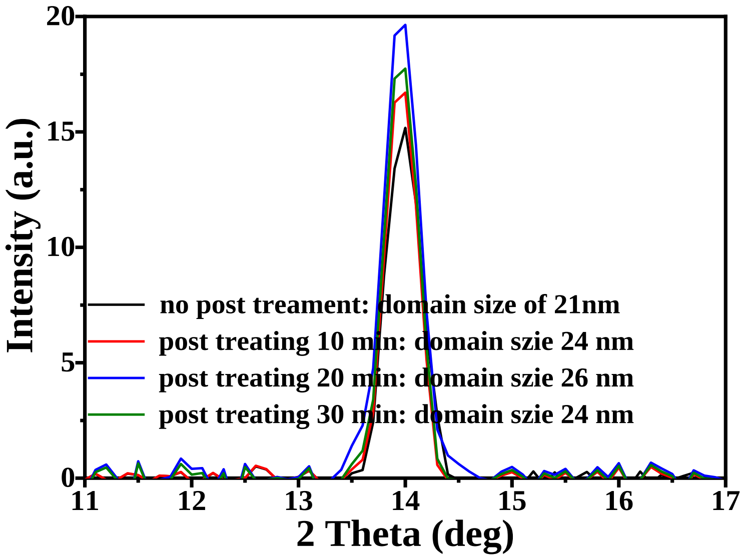

Figure S9: (100) diffraction peaks of perovskite film without post treatment and with water vapor post treated for different time.

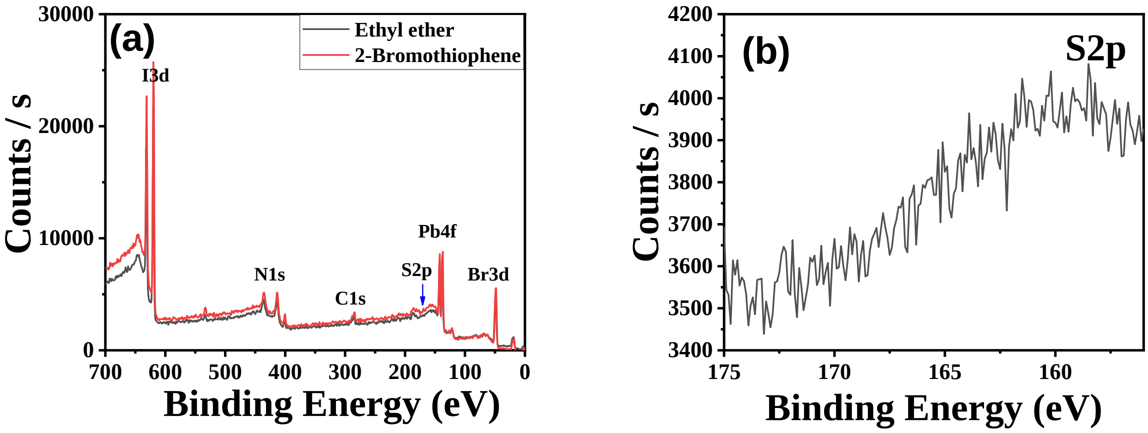

Figure S10: XPS spectra of perovskite (a) films prepared using ethyl ether or 2-bromothiophene as anti-solvent. (b) chemical scan (S2p) of perovskite prepared using 2-bromothiophene .

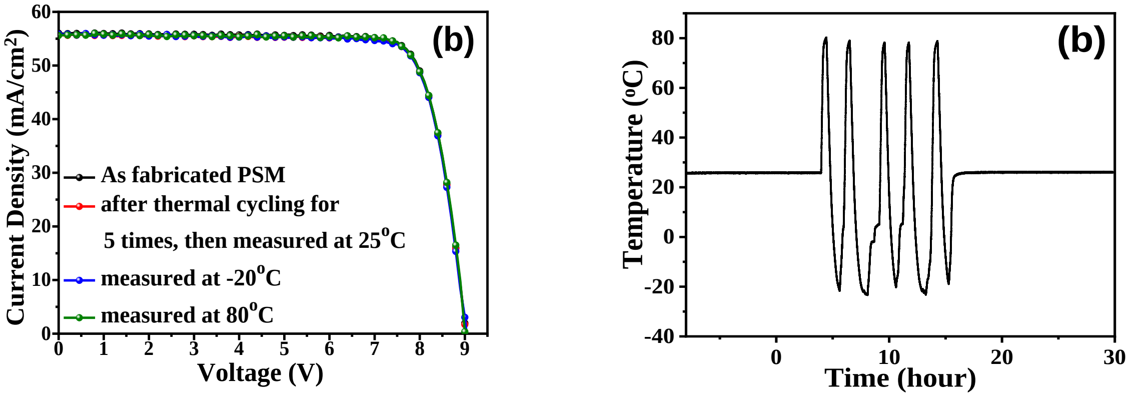

Figure S11: (a) *I-V* curves of the highest- efficiency PSMs as prepared, after thermal cycling for 5 time and measured at 25°C, -20°C and 80°C. (b) the temperature record in thermal cycling between -20°C and 80°C.

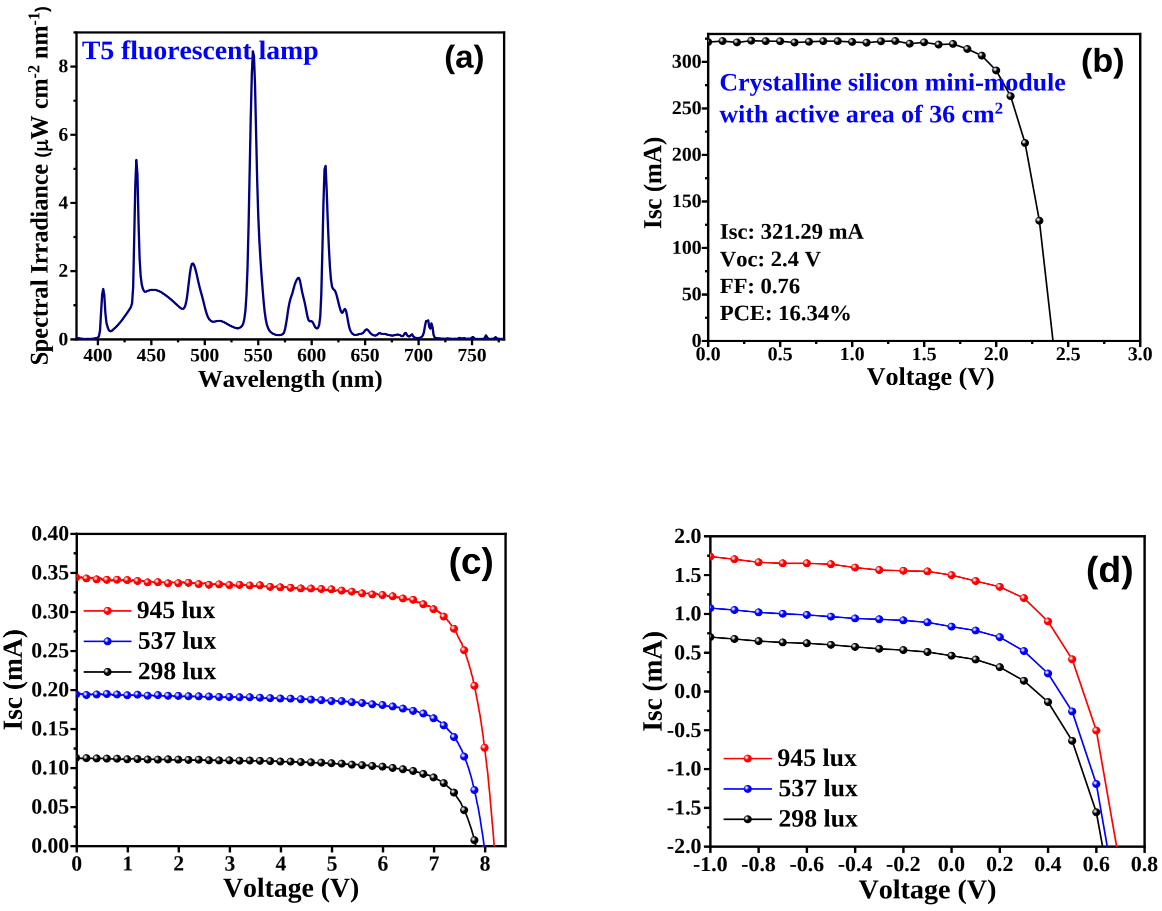

Figure S12: (a) the spectral irradiance of the T5 lamp used in indoor light study. (b) I-V curve and photovoltaic parameters of the crystalline silicon solar mini-module. (c) I-V curves of the high-efficiency PSM under T5 lamp illuminated at three light intensity. (d) I-V curves of the crystalline silicon solar mini-module under T5 lamp illuminated at three light intensity.
